# Granulomatous Myositis Associated with Extremely Elevated Anti-striated Muscle Antibodies in the Absence of Myasthenia Gravis

**DOI:** 10.7759/cureus.6981

**Published:** 2020-02-13

**Authors:** Sreenath Meegada, Hamza Akbar, Suman Siddamreddy, David Casement, Rajanshu Verma

**Affiliations:** 1 Internal Medicine, The University of Texas Health Science Center/Christus Good Shepherd Medical Center, Longview, USA; 2 Medicine, University of Texas at Dallas, Dallas, USA; 3 Internal Medicine, Baptist Health Medical Center, North Little Rock, USA; 4 Internal Medicine, Allina Health Shoreview Clinic, Shoreview, USA; 5 Gastroenterology, University of Tennessee Health Science Center, Memphis, USA

**Keywords:** myositis, granulomatous, myasthenia, proximal muscle weakness

## Abstract

Granulomatous myositis is a rare disease that predominantly results in proximal muscle weakness in the upper and/or lower extremities. As it can resemble other inflammatory myopathies, it is important to obtain a muscle biopsy to make the underlying diagnosis. We report the first case of granulomatous myositis associated with extremely elevated anti-striated muscle antibodies in a 69-year-old Caucasian woman. Granulomatous myositis has been associated with various autoimmune, infectious, rheumatologic, vasculitis, and oncologic disorders, and several antibodies have previously been reported to be associated with it. However, to the best of our knowledge, this is the first report where extremely elevated anti-striated muscle antibodies were found to be associated with granulomatous myositis in the absence of myasthenia gravis. The treatment of granulomatous myositis revolves around the use of corticosteroids, steroid-sparing immunosuppressive agents, and newer biologics.

## Introduction

Granulomatous myositis (GM) is a rare disorder affecting the proximal (and occasionally distal) muscles of the upper and lower extremities. Dysphagia can also be seen. GM is most commonly associated with sarcoidosis; however, several other conditions, such as Crohn's disease, tuberculosis, brucellosis, lymphoma, syphilis, thymoma/myasthenia gravis, primary biliary cirrhosis, Wegener’s granulomatosis, rheumatoid arthritis, and systemic sclerosis, can also result in GM. Though biochemical and serologic testing along with imaging helps rule in or rule out a particular diagnosis, the role of a muscle biopsy is paramount in confirming the diagnosis of granulomatous (sarcoid) myopathy [1]. Non-caseating granuloma formation with the presence of multinucleated giant cells is the hallmark of this disease.

## Case presentation

A 69-year-old Caucasian woman presented with complaints of left elbow pain for one week and bilateral thigh/calf pain for two weeks. She denied any pain at rest but her arms and legs started to ache on moving or climbing stairs. There was no associated trauma. On examination, she was found to have left lateral epicondylitis/tendinitis and was recommended naproxen and ice packs. Her atorvastatin, which she had taken at the same dose for the last 20 years, was held for a week, as rhabdomyolysis was suspected. A week later, she started noticing poor oral intake, hypoglycaemic episodes, dry mouth, constipation, occasional shortness of breath, palpitations, and left arm weakness. The primary care physician checked creatine kinase, electrolytes, and thyroid-stimulating hormone (TSH) and found that creatine kinase (CK) was elevated at 1259 IU/L (normal: < 168), magnesium 1.4 mg/dl (normal: 1.6-2.6), and TSH 30.68 UIU/ml (normal: < 5). Baseline TSH one year ago was 2.85 UIU/ml. Calcium corrected for albumin was found to be elevated at 15.0 mg/dl (normal: 8.5-10.5). The patient was admitted to the hospital for hydration and the correction of hypercalcemia and evaluation of her left arm weakness and other symptoms. She was asked to stop taking her calcium supplements (600 mg of calcium and 200 IU of vitamin D daily). Hypomagnesaemia was thought to be due to hypercalcemia and hydrochlorothiazide use. The electrocardiogram showed sinus tachycardia.

She had a past medical history of hypertension, type 2 diabetes mellitus, osteopenia, hypothyroidism (Hashimoto thyroiditis), vulvar dysplasia (had undergone resection for vulvar intraepithelial neoplasia 1), ocular hypertension, constipation, and low back pain. She has had a total abdominal hysterectomy with bilateral salpingo-oophorectomy for endometriosis, breast reduction surgery, previous dilation and curettage, right thyroid lobectomy, and appendectomy. She used to work as a receptionist, was a non-alcoholic, and smoked cigarettes for six years (6-7/day) in her twenties. The family history was significant for coronary artery disease, stroke, hypertension, hyperlipidemia in the father, and coronary artery disease and diabetes in the mother. She had been taking baby aspirin, glyburide-metformin twice daily, 70/30 insulin, levothyroxine, lisinopril-hydrochlorothiazide, multivitamin, and vitamin B6 100 mg daily. Her medications for diabetes were held due to poor oral intake and ensuing episodes of hypoglycemia, and she was started on magnesium supplementation.

Vital signs in the hospital showed a heart rate of 111 beats/min, blood pressure 120/87 mmHg, respiratory rate 16/min, temperature 97.8F, and oxygen saturation 99% on room air. Physical examination was unremarkable except for mild bilateral sensorineural hearing loss, difficulty using the left arm due to weakness, diminished handgrip, and finger-nose test incoordination.

The initial head computed tomography (CT) scan was negative for hemorrhagic stroke, tumor, or space-occupying lesion. Intravenous hydration with normal saline was started and tests to evaluate for hypercalcemia, palpitations, dyspnea, and muscle weakness were ordered. TSH was 11.91 UIU/ml, CK had improved to 717 IU/L, aldolase was elevated at 91.4 U/L (normal: < 7.7), parathyroid hormone (PTH) was 9.9 pg/ml (normal: 14.5-87.1), parathyroid hormone-related peptide (PTHrP) was 0.4 pmol/L (normal: < 2.0), prolactin was 8.1 ng/ml (normal: 5.2-26.5), and serum protein electrophoresis (SPEP) showed trace biphasic monoclonal protein immunoglobulin G (IgG) Kappa specificity with no free light chains. IgG and IgA levels were normal, IgM was low at 39 mg/dl (normal: 45-300), lactate dehydrogenase (LDH) was 286 IU/L (normal: 125-220), 25-OH vitamin D was 21.7 ng/ml (normal: 30-80), and 1,25 (OH)2 vitamin D was elevated at 145 pg/ml (normal: 18-78). Erythrocyte sedimentation rate (ESR) was 27 mm/hr (normal: <31) and C-reactive protein (CRP) was 1.1 mg/dl (normal < 0.5). Testing for hepatitis B and C was negative.

**Figure 1:**
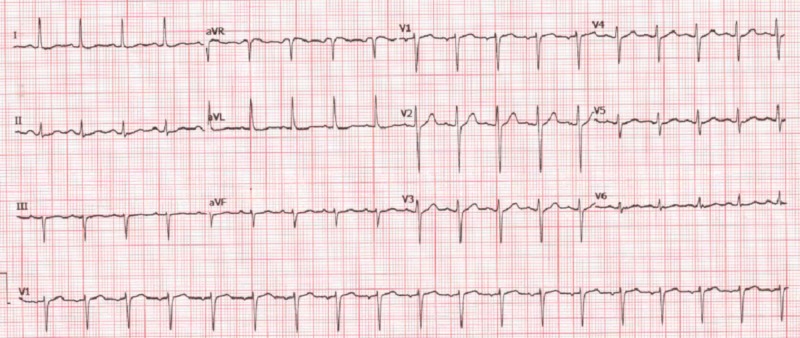
EKG showing sinus tachycardia EKG: electrocardiogram

Anti-nuclear antibody (ANA), anti-smooth muscle antibody, anti-Sm/RNP antibody, anti-Sm antibody, SS-A, SS-B, anti-Jo-1, anti-Scl 70, and anti-neutrophilic cytoplasmic antibodies (c-ANCA/p-ANCA) were all negative. Echocardiogram showed an ejection fraction of 60%-65% without any valvular abnormalities. The chest radiograph was normal. Electrocardiogram (EKG) showed sinus tachycardia (Figure 1).

She was given subcutaneous calcitonin and intravenous pamidronate for hypercalcemia. Given her generalized malaise and fatigue, the morning cortisol level and adrenocorticotropic hormone (ACTH) stimulation test were checked to evaluate for adrenal insufficiency, which came back normal.

**Figure 2:**
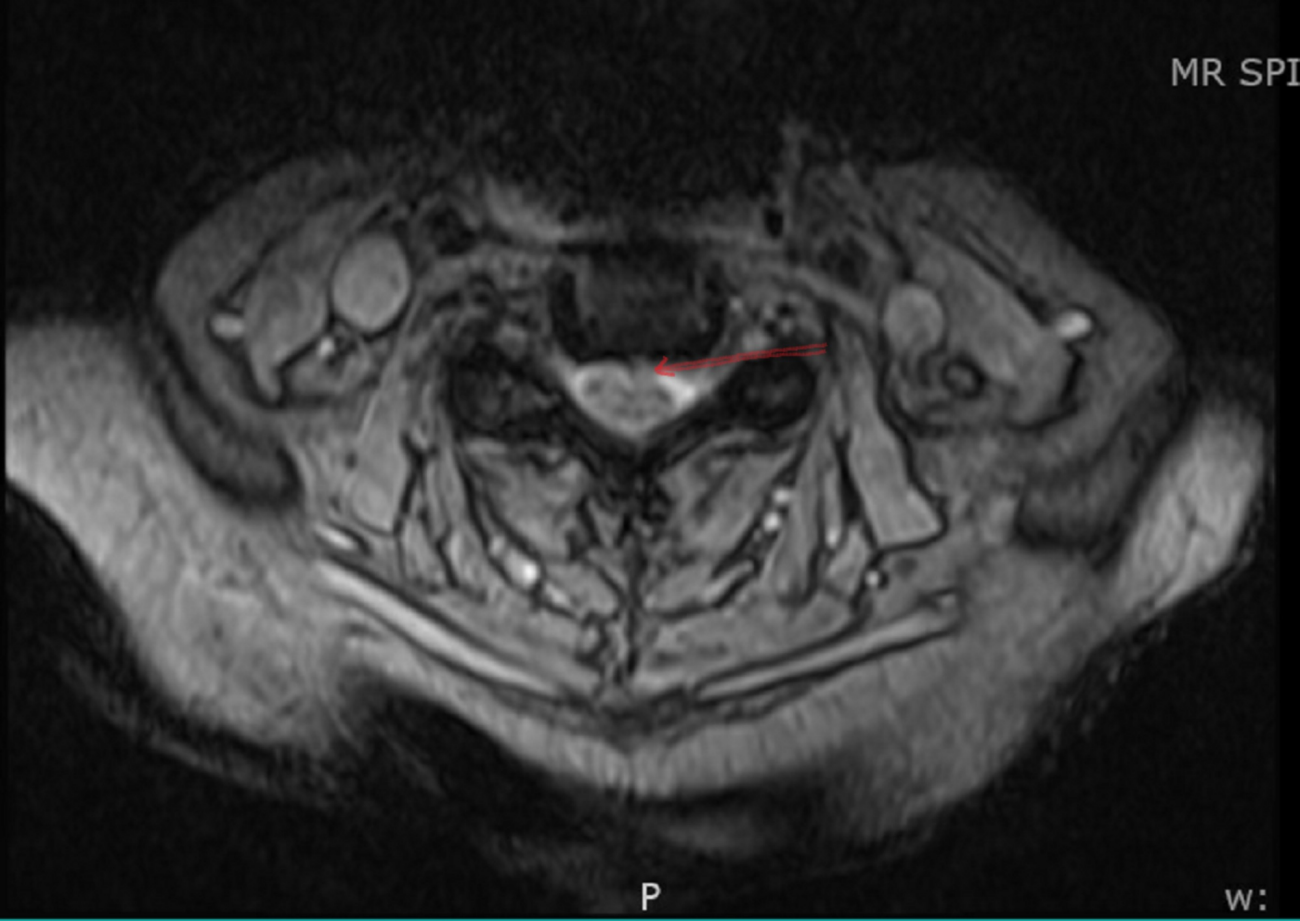
MRI cervical spine showing left side C5-C6 foraminal narrowing (arrow pointing)

Given concern for myositis and left arm weakness, a neurologist consult was called. The patient was found to be areflexic and to have proximal muscle weakness in the legs and distal weakness in the hands. MRI brain was unremarkable and MRI of the cervical spine showed marked right C4-C5 foraminal narrowing and marked left C5-C6 foraminal narrowing (Figure 2).

Electromyography (EMG) and muscle biopsy were recommended by the neurologist. Concern was raised about possible paraneoplastic syndrome v/s myopathy.

The patient was seen by physical and occupational therapy for rehabilitation and paraneoplastic panel was sent. Elevated 1,25 (OH)2 vitamin D pointed towards aetiologies such as sarcoidosis, other granulomatous diseases, and occult malignancies. A CT scan of the chest, abdomen, and pelvis showed a calcified right middle lobe granuloma but was otherwise negative. No thymoma was seen. A granuloma was noticed in the spleen. The serum angiotensin-converting enzyme (ACE) level was < 5 U/L (normal: 8-53); however, the patient had been on ACE inhibitor (lisinopril).

Electromyography (EMG) showed extensive abnormal spontaneous activity throughout, with a mix of myopathic and neuropathic features (Tables 1-2).

**Table 1:** Abnormal spontaneous activity throughout with a mix of myopathic and neuropathic features NCS: nerve conduction studies

Motor NCS	NERVE/SITES	LAT. ms	AMP. 1-2 mV	DIST. cm	VEL. m/s
	L MEDIAN - APB				
	Wrist	3.65	1.8	6.5	
	Elbow	7.4	1.6	20	53.3
	L ULNAR - ADM				
	Wrist	2.65	3.9	7	
	B.Elbow	5.95	1.6	16	48.5
	A.Elbow	17.05	0.3	11	9.9
	L COMM PERPNEAL - EDBx2				
	Ankle	NR	NR	9	
	Pop. Fossa	NR	NR	33	NR
	L TIBIAL MALLEOLUS - AH				
	Ankle	6.85	2.9	10	
	Pop. Fossa	14.9	2.7	33	41
Sensory NCS	NERVE/SITES	PEAK ms		AMP.1-2 µV	
	L HAND- SENSORY NERVES				
	Median, Antidrom II	3.00		28.0	
	Ulnar, Antidrom V	2.70		24.6	
	Median Palmar, Ortho	2.00		58.3	
	Ulnar Palmar, Ortho	1.70		22.0	
	Superfic Radial, Wr	2.45		17.0	
	L SURAL				
	Calf 14	3.60		2.8	
	L SUP PERPNEAL x2				
	Lat Leg	NR		NR	

**Table 2:** Abnormal spontaneous activity throughout with a mix of myopathic and neuropathic features EMG: electromyography

F Wave	NERVE	CURS 1 ms									
	L. ULNAR	29.35									
	L. TIBIAL MALLEOLUS	59.8									
EMG Summary Table		SPONTANEOUS				MUAP				RECRUITMENT	COMMENT
		Fib	PSW	Fase	IA	Number	Amp	Dur.	PPP	Rate	Type
	L. DELTOID	None	Norm	None	Norm	Norm	Norm	Norm	Norm	Norm	None
	L. TRICEPS	2+	2+	None	Norm	Incr	Norm	Norm	Poly	Norm	None
	L. BICEPS	None	Norm	None	Norm	Norm	Norm	Norm	Norm	Norm	None
	L. BRACHIORAD	2+	2-	None	Norm	Incr	Norm	Norm	Poly	Norm	None
	L. PRON TERES	2+	2+	None	Norm	Incr	Dec	Dec	Poly	Norm	None
	L. IST DORSINTEROS	2+	2+	None	Norm	Incr	Dec	Dec	Poly	Norm	None
	L. FLEX CARPIULN	2+	2+	None	Norm	Norm	Norm	Norm	Poly	Norm	None
	L. ABD DIG MIN	2+	2+	None	Norm	None					Can't Contract
	L. ABD POLL. BREV	2+	2+	None	Norm	Norm	Dec	Dec	Norm	Norm	None
	L. QUADRICEPS	3+	3+	None	Norm	SI Reduc	Incr	Dec	Poly	Norm	None
	L. ADD MAGNUS	3+	3+	None	Norm	Norm	Norm	Norm	Norm	Norm	None
	L. ILIOPSOAS	2+	2+	None	Norm	Norm	Norm	Norm	Norm	Norm	None
	L. TIB ANTERIOR	2+	2+	None	Norm	Norm	Norm	Norm	Norm	Norm	None
	L. GASTROC (MED)	2+	2+	None	Norm	Norm	Norm	Norm	Norm	Norm	None
	L. EXT DIG COMM	2+	2+	None	Norm	Incr	Norm	Norm	Poly	Norm	None
	L. EXT INDICIS	2+	2+	None	Norm	None	-	-	-	-	Can't Contract

The paraneoplastic panel showed negative ANNA-1, 2, 3, AGNA-1, PCA-1, 2, PCA-TR, amphiphysin antibody, P/Q type calcium channel antibody, N-type calcium channel antibody, acetylcholine receptor ganglionic neuronal antibody, neuronal V-G K+ channel antibody, and CRMP-5 IgG. The striated muscle antibody was highly positive 1:1966080 (normal: < 1:60) and the acetylcholine receptor (muscle) binding antibody was positive 0.15 nmol/L (normal: < 0.03). As a result of the antibody pattern seen on the paraneoplastic panel, other tests to confirm myasthenia gravis were done.

**Figure 3:**
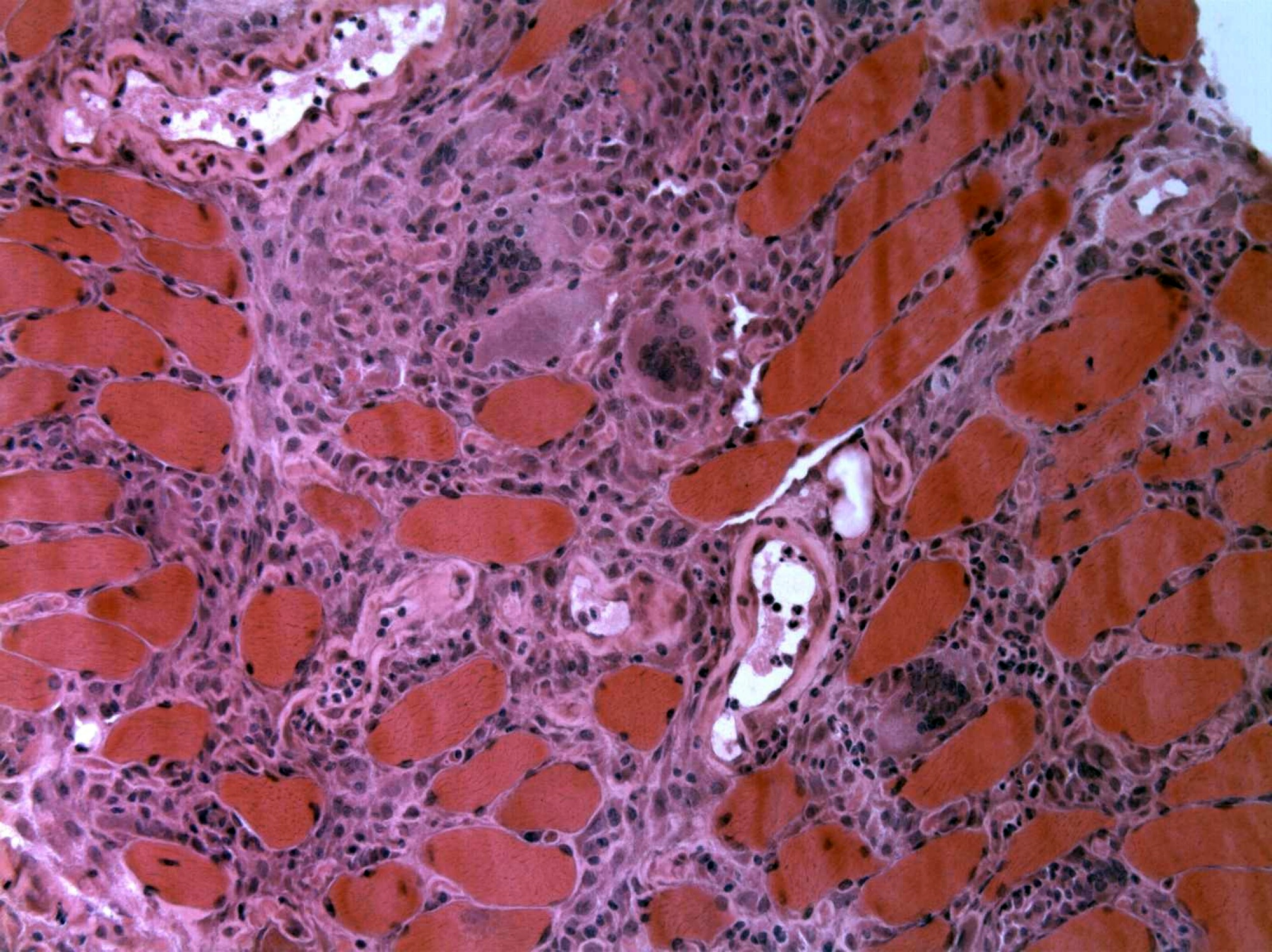
H&E stain of muscle biopsy showing inflammatory infiltrate consisting of lymphocytes, histiocytes, and multinucleated giant cells with type II fiber atrophy consistent with a diagnosis of sarcoid myopathy or any other granulomatous inflammatory myopathy H&E: hematoxylin and eosin

Right quadriceps muscle biopsy showed an inflammatory infiltrate consisting of lymphocytes, histiocytes, and multinucleated giant cells with type II fiber atrophy consistent with a diagnosis of sarcoid myopathy or any other granulomatous inflammatory myopathy (Figure 3).

Inclusion body myositis (IBM) would clearly be a close differential diagnosis owing to its similar clinical presentation (pattern of proximal muscle distribution, associated dysphagia, hand/finger flexor involvement) and creatine kinase elevation. Typically, IBM runs a more prolonged course (>5 yrs) than sarcoid myositis, which presents acute/subacutely. Histopathologic details help differentiate the two where IBM shows endomysial inflammation, CD8+ T lymphocytes, increased major histocompatibility complex class I (MHC-I) staining, cyclooxygenase (COX)-negative fibers, rimmed vacuoles, p62 or TDP-43 positive protein accumulation (amyloid), or 15 to 18 nm filaments whereas granulomatous (sarcoid) myositis shows the presence of granulomas and multinucleated giant cells.

The patient was discharged to a skilled nursing facility for rehabilitation. Assuming the presence of myasthenia, the primary care physician started her on pyridostigmine, which failed to improve her symptoms and so it was discontinued. A week into the skilled nursing facility stay, the patient began having symptoms of dysphagia and had to be readmitted to the hospital for hydration, swallow evaluation, and correction of hypercalcemia. Swallowing evaluation was abnormal so a percutaneous endoscopic gastrostomy (PEG) tube was inserted to provide nutrition with tube feeds. The patient’s dysphagia was thought to be associated with sarcoid/granulomatous myopathy as well.

In order to ascertain the diagnosis of possible concurrent myasthenia gravis along with sarcoid myopathy, she was referred to a neurologist at a tertiary care center. Further testing showed elevated acetylcholine receptor (muscle) modulating antibody at 58% (normal < 21) and elevated acetylcholine receptor (muscle) blocking antibody at 53% (normal <15). The tertiary center neurologist found a significant weakness in the proximal muscles of upper extremities, weakness in the intrinsic muscles of the hand, and reduced reflexes but no diplopia, ptosis, or nasal speech. Both edrophonium testing and repetitive nerve stimulation studies were negative. Nerve conduction studies and repeat EMG showed evidence of a moderately severe myopathy most prominent in the proximal musculature with electrophysiologic correlates of fiber splitting, myonecrosis, or vacuolization in most muscles. No evidence of large fiber peripheral neuropathy or a neuromuscular junction defect was found. Given the absence of clinical presentation, EMG, and nerve stimulation studies, MG was ruled out and associated antibodies were thought to be possibly paraneoplastic in nature. A positron emission tomography (PET) scan was done to look for underlying malignancy, which showed diffuse non-focal increased activity was seen in muscles. Rheumatology was consulted for the management of sarcoid or granulomatous myositis. She was started on 1 mg/kg (75 mg) of prednisone a day, which was gradually tapered down to 20 mg per day over eight weeks. Her muscle weakness and dysphagia improved somewhat over the course of the next two months. In order to minimize the long-term side effects of corticosteroid use, methotrexate was added to the prednisone taper to treat sarcoid myopathy. Over the course of the next four months, her myositis, muscle weakness, dysphagia, and strength had improved significantly. While being on methotrexate, she started to develop transaminitis (2-3x upper limit of normal) along with mild thrombocytopenia. These were thought to be complications of methotrexate treatment. An ultrasound of the liver was done, which showed fatty liver.

Two months later, she was readmitted to the hospital for shortness of breath, which was attributed to ascites secondary to portal hypertension. CT abdomen showed moderate ascites, raising concern for developing cirrhosis and portal hypertension. Anti-smooth muscle antibody to detect autoimmune hepatitis came back negative. Due to her declining condition, the patient and family members chose hospice care, as she did not want to continue to undergo further treatment and testing for her illnesses. The patient was discharged to home with hospice. Three weeks later, she died at home. An autopsy was performed, which showed cirrhosis (though there was no evidence of sarcoidosis in the liver), coronary artery disease, and lymphocytic thyroiditis with nodular hyperplasia. Her cirrhosis was attributed to nonalcoholic steatohepatitis.

## Discussion

Granulomatous/sarcoid myositis is a rare disorder that can involve skeletal muscles throughout the body. Though muscles are involved in sarcoidosis in up to 20%-80% of cases, most of these cases are asymptomatic [1]. Symptomatic muscle involvement in sarcoidosis is rare and ranges from 0.5%-2.3% [1-2]. Historically, muscular involvement in sarcoidosis has been divided into three distinct forms: chronic myopathy subtype (with atrophy and muscle wasting or occasional pseudohypertrophy), palpable nodules, and acute myositis subtype. It appears that the non-nodular form of the disease tends to occur more commonly in females than in males and typically affects them in the fifth to seventh decades of life though cases outside this age range have been reported as well [3]. Proximal muscles are typically involved in sarcoid myositis although distal muscle involvement may also be seen. Dysphagia has been associated with sarcoid myositis as seen in our patient [1,4]. On clinical examination, deep tendon reflexes are usually diminished or clinically absent [5]. EMG shows a myopathic pattern with spontaneous discharge activity [1]. MRI has been suggested as a useful adjunct in diagnosing sarcoid myositis, whereas gallium scintigraphy may be helpful in diagnosing the nodular form of the disease [6-7]. A PET scan has been used to help support the diagnosis of granulomatous myositis as well [8]. Serum or cerebrospinal fluid (CSF) ACE levels were not elevated in our patient; however, she had been taking ACE inhibitors. Serum ACE levels have been found to be sensitive markers in the pulmonary form of sarcoidosis though they may not be reliably elevated in other forms such as sarcoid myopathy [3]. Serum creatinine kinase (CK) levels may be elevated in patients with acute myositis but may be normal in the chronic myopathic subtype [3]. Muscle biopsy is of paramount importance in the diagnosis of inflammatory myopathies. It helps distinguish sarcoid myositis from other mimickers such as inclusion body myositis. Type II fiber atrophy has previously been reported in sarcoid myositis [9]. Though in previous reports, patients with type II fiber atrophy benefitted from ongoing steroid use, prednisone had a probably marginal benefit in our case [10].

The treatment of sarcoid myopathy revolves around one or the other forms of immunosuppression. Due to only a couple of hundred reported cases in the literature, no randomized trials exist on its treatment options. Management principles have been borrowed from the treatment of systemic sarcoidosis and involve the use of corticosteroids, methotrexate, azathioprine, intravenous immunoglobulins (IVIGs), infliximab, adalimumab, etanercept, and other biologics. Response to these drugs is variable and fraught with their complicating side effects. Though steroids are usually the first line and the most commonly used agents, methotrexate seems to be more effective in treating sarcoid myositis than prednisone [11]. Most patients require treatment though rare, spontaneous remission has also been reported [12]. There have also been anecdotal reports of the use of IVIG for refractory cases, anti-tumor necrosis factor (TNF) therapies, and the use of newer biologics in the treatment of sarcoid myopathy [13].

A separate disease entity named sarcoid-like myositis, where patients have severe hypercalcemia, the absence of bilateral hilar adenopathy, and steroid responsiveness has been proposed apart from sarcoid myositis though patients in this cohort universally had normal CK levels, which was not seen in our case [14]. Thus, it is difficult to classify our patient under the category of sarcoid-like myositis.

Though the co-occurrence of granulomatous myositis in a patient with myasthenia gravis has previously been described and granulomatous myositis has been associated with elevated antithyroglobulin/antithyroid peroxidase antibodies and with anti-mitochondrial antibodies, to the best of our knowledge, the association of granulomatous (sarcoid) myositis with elevated myasthenia antibodies (in a patient who does not have myasthenia) has not been previously reported [15-18]. Though myositis-specific antibodies have long been known, the etiology, clinical significance, and mechanism of production of MG antibodies in granulomatous (sarcoid) myositis warrant further research.

## Conclusions

GM is a rare disease involving the skeletal muscles of the body. Diagnosis is made with the help of a muscle biopsy. Manifestations include weakness of involved muscles (upper/lower extremities, head and neck muscles, muscles of respiration, and others). The treatment of GM involves some form of immunosuppression, e.g., corticosteroids, methotrexate, IVIG, anti-TNF therapy, and other biologics. GM has been associated with several antibodies; however, after conducting an extensive literature search, to the best of our knowledge, it appears that its association with extremely elevated anti-striated antibodies in the absence of concurrent MG has never been reported.
